# Inflammatory Nasal Swelling due to *Leishmania tropica*

**DOI:** 10.1155/2021/3801949

**Published:** 2021-12-27

**Authors:** Hajiba Fellah, Maryam Hakkour, Bouchra Delouane, Asmae Hmamouch, Abdelhakim Bouyahya, Faiza Sebti, Abderrahim Sadak

**Affiliations:** ^1^Laboratory of Biodiversity, Ecology and Genome, Faculty of Sciences, Mohammed V University in Rabat, Rabat, Morocco; ^2^National Reference Laboratory of Leishmaniasis, National Institute of Hygiene, Rabat, Morocco; ^3^Laboratory of Microbial Biotechnology, Sciences and Techniques Faculty, Sidi Mohammed Ben Abdellah University, Fez, Morocco; ^4^Laboratory of Human Pathologies Biology, Department of Biology, Faculty of Sciences, and Genomic Center of Human Pathologies, Faculty of Medicine and Pharmacy, Mohammed V University in Rabat, Rabat, Morocco

## Abstract

Since its discovery in the 19th century, cutaneous leishmaniasis has been a major public health problem, especially with the appearance of more and more unusual cases of cutaneous lesions due to this parasite. Indeed, the present study joins the previous studies and describes a typical case of a nasal lesion due to *Leishmania* infection. This is a 20-year-old young man, with no particular pathological history, from an epidemic focus who presented with inflammatory nasal swelling similar to a mucocutaneous form. However, the X-ray data showed that no lysis of the bones proper to the nose was detected and no damage to the underlying mucosa was observed. Nevertheless, the parasitological diagnosis confirmed the presence of amastigotes, and the results of the molecular study showed that the electrophoretic profile was comparable to that of *L. tropica*. After diagnosis and confirmation, treatment with meglumine antimonate at the rate of two ampoules/injection (one ampoule = 5 ml) of antimony salt for one month was administered intramuscularly with favorable outcome. Atypical forms of cutaneous leishmaniasis constitute a rare and unusual entity often leading to diagnostic delay. For this, the clinical examination must take into account both exceptional presentations of *Leishmania* infection, in particular in subjects living or having stayed in an endemic area, in order to ensure appropriate and early treatment.

## 1. Introduction

Leishmaniases include several clinical forms depending on the pathogen, ranging from skin ulcers and nodules to mucosal disorders with more serious lesions, up to visceral infections that damage internal organs [1]. This multiplicity of clinical pictures results both from a wide variety of species and from the variation in the immune response of the infected host.

In Morocco, cutaneous-mucous leishmaniasis has never been declared. However, cutaneous leishmaniasis (CL) constitutes a major national public health problem. It is caused by three *Leishmania* species, namely, *Leishmania major* (*L. major*), *Leishmania tropica* (*L. tropica*), and *Leishmania infantum* (*L. infantum*) [2]. Nevertheless, each species is characterized by a different clinical polymorphism [3].

In order to examine the epidemiological and clinical particularities of CL, a rare form of facial lesion has been studied and described. Thus, a molecular study was developed to determine the species responsible for this unusual lesion.

## 2. Case Description

**Figure 1:**
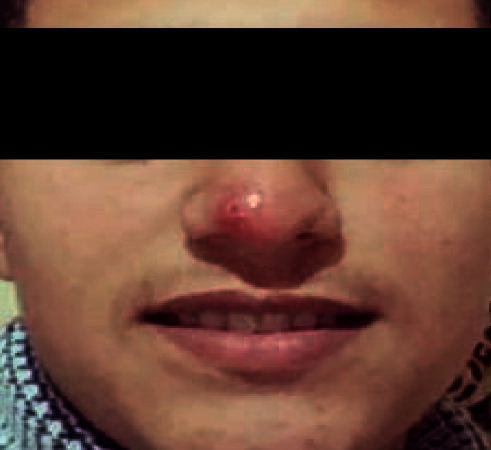
Onset lesion of the nasal infection.

**Figure 2:**
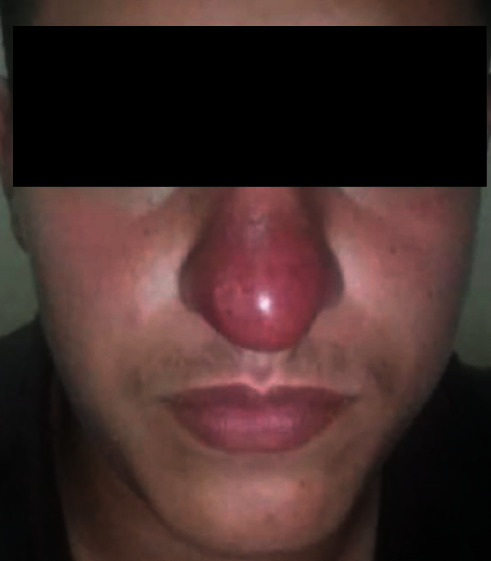
Erythematous appearance covering the entire lobe of the nose.

This is a case of a 20-year-old patient with no particular pathological history, originally from the province of Taounate and moving between this province and the province of Meknes. However, he was infected in the province of Taounate, especially in the rural sector of Ghafsai where he carries out his agricultural activities. He consulted for a skin lesion in the nose. The disease started around May 2018 with a small localized lesion (Figure 1). Four months later, the lesion has spread over the entire lobe of the nose and appeared erythematous (Figure 2).

**Figure 3:**
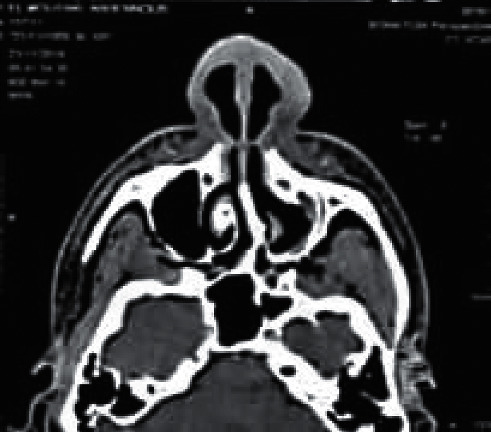
CT scan of the facial mass: no destruction of the nasal septum.

**Figure 4:**
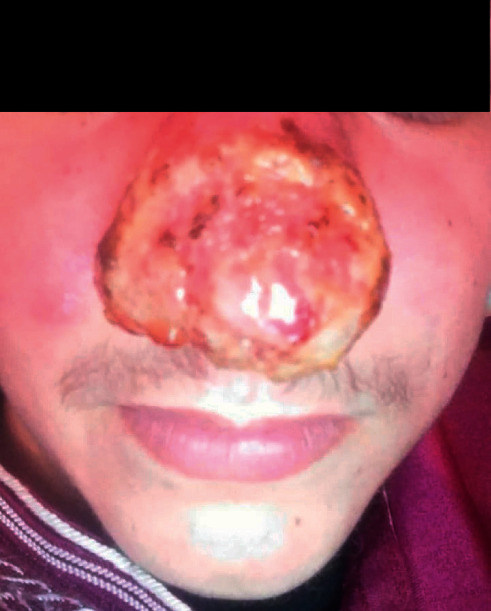
Inflammatory nasal swelling due to *Leishmania tropica*.

In front of all these clinical elements, the patient had received antibiotic treatment without improvement. After that, several diagnoses were suggested, namely, sarcoidosis and leishmaniasis, and the observational clinical examination had suspected cutaneous mucosal leishmaniasis. The pathological anatomy results performed in a private center showed an inflammatory reaction composed of mononuclear cells, sometimes epithelioid with a vaguely granulomatous arrangement without caseous necrosis. However, these same results showed the absence of a formal argument for sarcoidosis as well as the absence of an obvious *Leishmania* body. As for the X-ray results, no lysis of the specific bones of the nose was detected and no damage to the underlying mucosa was observed (Figure 3). One month later, the patient was presented to the national leishmaniasis reference laboratory at the national institute of hygiene in order to confirm the diagnosis of CL. Clinical examination revealed an ulcerative, infiltrated, and poorly limited erythematous placard (Figure 4).

**Figure 5:**
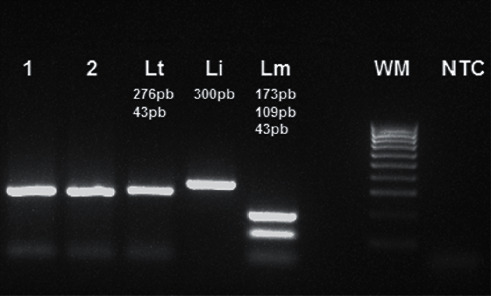
PCR-RFLP technique used for identification of *Leishmania* species (enzymatic digestion of PCR fragments by Mn1-I).

The microscopic diagnosis from skin samples showed the presence of *Leishmania* amastigotes within the macrophages. These results were confirmed by molecular diagnosis using the ITS1 PCR-RFLP technique. In addition, the enzymatic digestion carried out by MN1-I enzyme made it possible to compare the sample received and the three *Leishmania* species circulating in Morocco, namely, *L. tropica*, *L. major*, and *L. infantum*. However, the results showed that the electrophoretic profile was comparable to that of *L. tropica* (Figure 5).

Lane 1-2: patient smears and lane WM: weight marker 100 bp. Positive controls: Lt, *L. tropica*; Li: *L. infantum*; Lm: *L. major*; and NTC: negative control. The electrophoretic profile of the strain isolated in the patient was comparable to the *L. tropica* profile.

Immediately after laboratory confirmation and after an X-ray of the healthy liver and lungs, treatment with meglumine antimonate (Glucantime®) was initiated according to the protocol described by the Ministry of Health, generally 20 mg of SB5+/kg, without exceeding 2 ampoules, for 3 weeks. For our patient, two ampoules/injection (one ampoule = 5 ml) of antimony salt were administered intramuscularly. Due to the extent and location of the lesion, the duration of treatment was one month.

**Figure 6:**
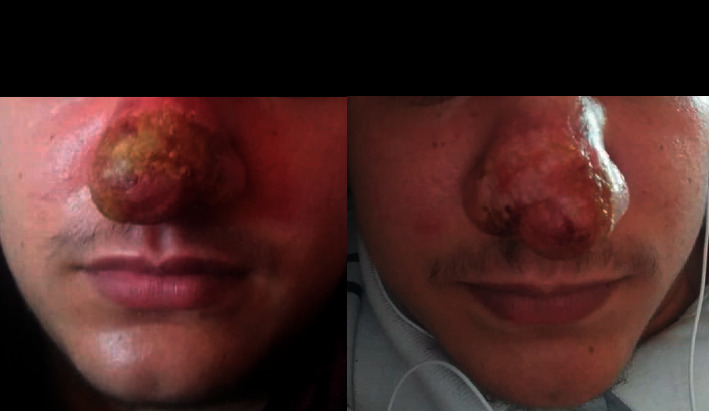
Response of the lesion to meglumine antimoniate treatment: one week after beginning of treatment.

**Figure 7:**
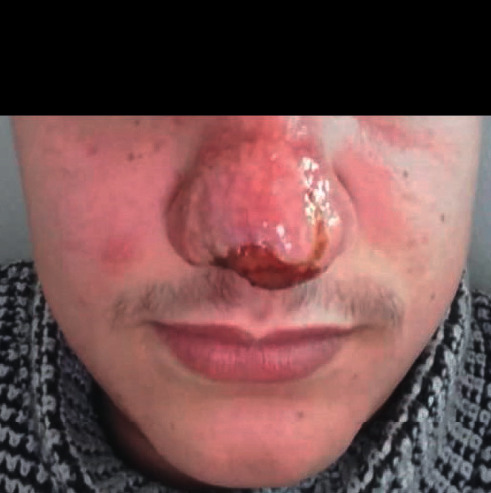
Response of the lesion to meglumine antimoniate treatment: 2 weeks after beginning of treatment.

**Figure 8:**
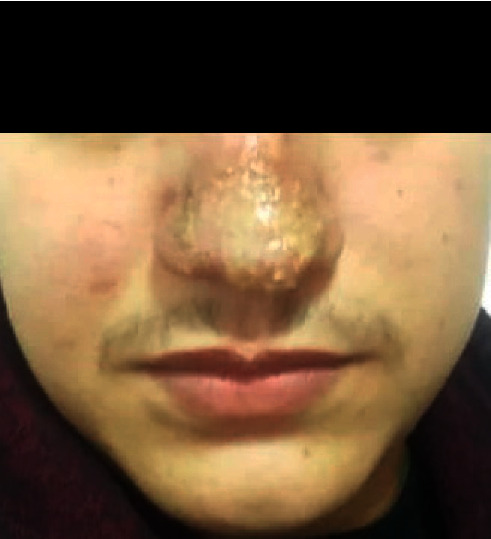
Response of the lesion to meglumine antimoniate treatment: stopping treatment.

The initiation of treatment had shown good tolerance and satisfactory results (Figure 6). A week later, a clear clinical improvement was noticed and the skin lesion healed completely without relapse (Figure 7). Nevertheless, the patient still retains partial healing of the lesion (Figure 8). However, cosmetic surgery was subsequently mandatory to remove the residue from the lesion. This was the consequence of late treatment because the patient did not come to the leishmaniasis reference center (INH) until after having consulted quite a few private centers.

## 3. Discussion

Leishmaniases are caused by a heterogeneous group of protozoan parasites belonging to the *Leishmania* genus which are responsible for many clinical presentations.

The epidemiology of the cutaneous form is complex due to intra- and interspecific variations in transmission cycles, reservoir hosts, sandfly vectors, and clinical manifestations, as well as the presence of multiple *Leishmania* species in the same geographical area which are phylogenetically distinct [4].

Mucocutaneous leishmaniasis is the most severe form of the disease, and it is mainly caused by the *L. braziliensis* complex. About diffuse cutaneous leishmaniasis, it is caused by *L. aethiopica*, and the Daman constitutes the main reservoir of the parasite.

In Morocco, neither of these two integumentary forms has been described so far, giving way to localized cutaneous leishmaniasis, which constitutes a major public health problem.

The study is the subject of a rare clinical appearance of a lesion caused by the *L. tropica* species. Indeed, this species has the largest geographical distribution in Morocco compared to the Maghreb countries. Its transmission can be both urban and rural [5–7]. In Morocco, leishmaniasis is a real public health problem. To deal with this pathology, a National Leishmaniasis Control Program has been established since 1997 [8]. The control measures for this program are as follows:

- Earlier detection and treatment of leishmaniasis cases (screening in schools and localities)
- Vector control action: chemical action (intradomiciliary insect control or in stables, caves, household refuse, and manure dumps); physical action (sanitation and hygienic disposal of household waste and collective hygiene); and individual protection (by impregnated mosquito nets)
- Action to control the reservoir through the fight against stray dogs and rodents through intersectoral collaboration
- Information, education, and communication (training and information of staff; awareness of the population; and associations)
- Intersectoral collaboration: the Ministry of the Interior (environmental sanitation and hygiene and fight against stray dogs); Ministry of Agriculture (rodent control and fight against stray dogs as part of the fight against zoonoses); and Ministry of Health (patient care)

Unusual lesions due to *L. tropica* or other *Leishmania* species are no longer surprising in both reservoirs and humans. The usual clinicopathological picture of human CL varies from erythematous papules to noduloulcerative forms. However, in various endemic countries, unusual forms of CL have been described including erysipeloid, lupoid, annular, mucocutaneous, chancriforms, acute paronychic, palmoplantar, fissure leishmaniasis, zosteriform, submaxillary subcutaneous nodule, leishmaniasis scarring, whiteness, discoid lupus erythematosus-like, eczematous, squamous cell carcinoma-like, verrucous, and panniculiticvariants [9–14].

The clinical picture of leishmaniasis depends not only on the infecting leishmaniasis species but also on the host's immune response, which is largely mediated by cellular immunity. This immunity actually leads to a wide variety of presentations in a geographically restricted area. The response may be associated with the resistance of the *Leishmania* strain to macrophages, defective macrophage function, decreased production of interferon, imbalance between TH1 and TH2 cells, decreased chemotaxis, or increased delayed hypersensitivity [11].

In addition to the immune status, other factors can affect the clinical picture including the number of parasites inoculated, the site of inoculation, the nutritional status of the host, old age, menopause with its hormonal changes, and use of oral steroids and even wound contamination with inorganic materials.

Coinfection with human immunodeficiency virus (HIV) can also alter the clinical picture of CL. Indeed, HIV infection has led to the development of atypical forms of VL with an increased incidence of skin involvement. Several forms of the skin lesions have been reported worldwide including papulonodular lesions, psoriasiform-like and dermatomyositis-like eruption, erythroderma, and polymyositis-like syndrome [15].

Our patient is male and younger than the described cases, which works against senile immune impairment. In addition, he does not suffer from any disease of the immune system, and no trauma has been described. However, he resides in a province known for its history of leishmaniasis, both the cutaneous and visceral form.

Usually lesions are seen on the exposed parts of the body. Several studies have described isolated cases of nasal involvement following CL. However, nasal leishmaniasis was considered infrequent. On the other hand, a study showed that 29% of cases of CL had a nasal infection and that young people, adolescents, and young adults were the most affected [11]. This nasal infection may be due to the fact that the nose is the most projected part of the face and its inability to avoid sandfly bites by pushing it away. However, this is the first time that such an aspect of lesion has been observed in the nose, whether at the institute level or at the provincial level.

Finally, due to the wide spectrum of nasal CL diseases in endemic areas, practitioner training is essential taking into account all lesions resulting from *Leishmania* infection which do not respond to appropriate conventional therapies.

## 4. Conclusions

Our observation shows an *L. tropica* infection with an unusual lesion in a young man with no particular pathological history. However, he resides in a province known for its history of being foci of cutaneous and visceral forms of leishmaniasis. Given these facts, the clinical examination must always take into account the unusual lesions resulting from infection with *Leishmania*, especially in subjects living or having stayed in the endemic area, in order to ensure appropriate and early treatment.

## Data Availability

The data used to support the findings of this study are included within the article.
